# Evaluating Vacuum and Steam Heat to Eliminate Pinewood Nematodes in Naturally Infested Whole Pine Logs

**DOI:** 10.2478/jofnem-2024-0038

**Published:** 2024-10-05

**Authors:** J. D. Eisenback, Z. Chen, M. White

**Affiliations:** Professor, School of Plant and Environmental Science, Virginia Tech; Research Scientist and Professor Emeritus, respectively, Virginia Tech Center for Unit Load Design, Virginia Tech, Blacksburg, VA 24061

**Keywords:** *Bursaphelenchus xylophilus*, environmentally friendly, forest health, fumigation, international trade, ISPM-15, lethal temperature, methyl bromide, phytosanitary treatments, pinewood nematode, *Pinus* spp., steam heat, survival stage, yellow pine trees, vacuum, whole logs

## Abstract

Pinewood nematodes threaten forest health and continue to interfere with international trade because they can be spread around the globe via nematode-infested wood. International Standards for Phytosanitary Measure (ISPM-15) requires that all pine wood be treated at 56°C for 30 min to ensure that all pests and pathogens are killed within sawn wood, whereas fumigation with methyl bromide is the currently approved practice and widely used in treating whole logs. A method of treatment that uses less energy and time or does not rely on environmentally damaging gases is urgently needed. Because vacuum with steam has shown promise in treating several different commodities, the purpose of this study was to use it to eradicate pinewood nematodes in whole logs. Three protocols were applied: 1) 48°C for 15 min., 2) 56°C for 30 min., and 3) 60°C for 60 min. The third protocol reduced the population to statistically zero; however, some samples contained at least 1 survivor. Unfortunately, these surviving nematodes increased in number one month after treatment, and one year later, they continued to reproduce in the wood. Therefore, this protocol needs to be further refined to remove pinewood nematodes completely. Explanation of the survival of individual nematodes within whole logs remains a matter for conjecture: 1) certain portions of the wood were somehow insulated from the heat and did not achieve the lethal temperature, and 2) survival stages may be able to survive temperatures that are deadly to the normal life stages.

## Introduction

Pinewood nematodes (*Bursaphelenchus xylophilus* (Steiner & Buhrer, 1934) Nickle, 1971) continue to cause significant interference with world trade and shipping (Espada *et al*. 2022). Native trees in North America are efficient hosts of this nematode but rarely become diseased or die. Pinewood nematodes are efficiently vectored by the pine sawyer beetles (*Monochamus* spp.) which feed on pine needles and release nematodes onto the cut surfaces of needles (Dwinell 1997; Kim *et al*. 2020). They then penetrate the tree and feed within the vascular tissues and rapidly reproduce by amphimixis. The nematodes become so numerous inside the host that they invade all the tissues, both dead and living. In susceptible trees, the nematodes block the movement of water in the xylem, causing the tree to rapidly wilt; this is called pine wilt disease (Mamiya 1983). The needles quickly turn from green to orange to brown in just a few (4–8) weeks.

After the tree dies, the nematodes continue to feed on plant tissues, but they can also feed on fungal hyphae which often invade the wood of the dead tree. There, they can continue to reproduce and “ramify the wood.” In North America, trees with high populations of pinewood nematodes in native tree species are not sensitive to the nematode and generally show no symptoms of infestation (Dwinell 1997). When trees are harvested, the logs may be free of nematodes, or they may house low to high populations of nematodes; however, nematodes that are present are able to increase in number even though the wood is dead. This phenomenon is problematic for international trade in whole logs since an undetected population of nematodes can increase to very high numbers during the long days of shipping (Dwinell 1997).

Unfortunately, tree species in Asia and Europe are very sensitive to this nematode which has become a major pest in the forests of China, Japan, South Korea, Portugal, and other countries (Kim *et al*. 2020). In China, pinewood nematode has killed more than 50 million trees causing 22 billion USD in losses since 1982 (Qiu 2013).

Because of the threat to native forest trees, solid wood packaging, including wood pallets leaving the United States, is subject to International Standards for Phytosanitary Measures (ISPM-15) compliance, which requires them to be treated at 56°C for 30 min. throughout, or fumigated with methyl bromide to ensure that all pests and pathogens are killed (Allen, 2013). A targeted pest of this protocol is the pinewood nematode. Uzunovik *et al*. (2013) confirmed that temperatures at or above 56°C for 1 minute in small wood samples using radio frequency heating is lethal to the pest.

Accepted phytosanitar y treatments for unsawn wood, such as logs for export, have relied significantly on methyl bromide fumigation and or debarking. Limitations in the use of methyl bromide have resulted in the search for alternative fumigants including sulfuryl fluoride, phosphine, and ethanedinitrile (Arbuzova *et al*. 2021). These trials have not resulted in an accepted replacement for methyl bromide without also debarking. Removing the bark from the log prior to shipment reduces the log value so it is to be avoided if possible. Although heat treatment is accepted as a fumigation alternative, the conventional, hot air oven methods are unacceptable for treating whole logs for two reasons: 1) very high temperatures are required for long time periods to treat the log, and 2) the resulting drying damages the logs by producing large splits (White *et al*. 2017).

Vacuum technology with the use of steam may eradicate the pinewood nematode without the harmful environmental effects of methyl bromide and without impact on log value. Furthermore, it uses less energy and is therefore more cost-effective than conventional atmospheric heat treatment methods. In combination, vacuum, and steam do not impact the quality of the wood and are much faster than conventional heat (Chen *et al*. 2023). It has been used successfully for treating several commodities, including cotton, seeds, spices, mushroom media, and other products (Kozempel *et al*. 2003; Shah *et al*. 2017). As a treatment for several pests and pathogens of logs, vacuum, and steam promises to be a reliable and effective treatment that may replace methyl bromide (Chen *et al*. 2017a; Chen *et al*. 2017b; Juzwik *et al*. 2019; Juzwik *et al*. 2021). The ability to kill pinewood nematodes in whole, naturally infested logs remain to be tested. The four objectives of this research were as follows: 1) to determine the effectiveness of vacuum steam to kill pinewood nematodes, 2) to decide the most efficient and efficacious schedule for treating naturally infested pine logs, 3) to record temperature profiles and times required for each treatment, and 4) to document any impact of the treatment on log quality.

## Materials and Methods

### Selection of infested logs

**Figure 1:**
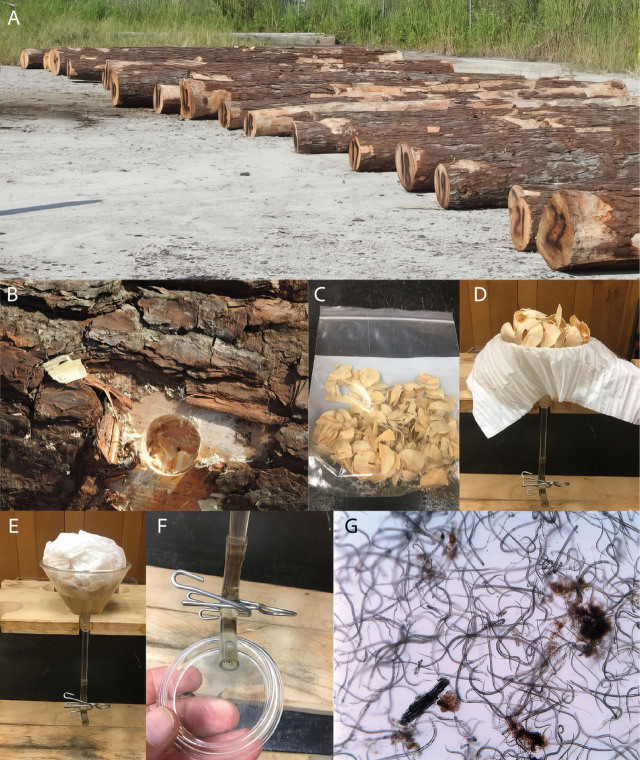
Sampling and extraction of the pinewood nematode (*Bursaphelenchus xylophilus* (Steiner & Buhrer, 1934) Nickle, 1971) from whole pine (*Pinus* spp.) logs. A) Thirty yellow pine (*Pinus* spp.) logs laid out in the woodlot and numbered, ready to be sampled for pinewood nematodes. B) Hole drilled in an area without bark with a 2.5 cm diameter Forstner drill-bit 2.5 cm in depth with chips collected and placed in a quart-size plastic bag (C) and kept on ice while in the field. C,D) Ten grams of wood chips were placed on a single sheet of facial tissue paper on a Baerman funnel filled with water and covered with the tissue. E,F) After 12–16 hours, 5ml of water was drained from the plastic tube and examined for nematodes with a dissecting microscope. G) Pinewood nematodes were tentatively identified by overall body shape and characteristic movement.

A woodlot in North Carolina was agreeable to testing for nematode-positive logs. Thirty logs were laid on the ground in a row and numbered with paint (Fig. 1A). One sample was collected from each log by drilling a 2.5cm diameter by 2.5cm deep hole with a Forstner drill bit in each log (Fig. 1B), and the wood shavings were placed in a plastic bag and labeled with the same number as the log (Fig. 1C). The location on the log where the sample was taken was near one end in an area that was free of bark. The collected wood shavings were stored on ice in a cooler after being sampled and during their transport back to the lab.

The wood chips were placed on a single layer of facial tissue that was put inside a 230ml Baermann funnel with 30ml of fresh bottled water (Figs. 1D,E). After 12 hr., the water near the bottom of the tube was drained into a Bureau of Plant Industry (BPI) dish (Fig. 1F) and observed with a dissecting microscope at 30× for the occurrence of pinewood nematodes (Fig. 1G). The first set of 30 logs was negative; however, in the second set, 27 out of 30 logs were positive. Twenty positive logs were selected and shipped to the campus of Virginia Tech in Blacksburg, VA, for vacuum and steam treatment in an attempt to rid them of pinewood nematodes.

### Vacuum and steam treatment

**Figure 2:**
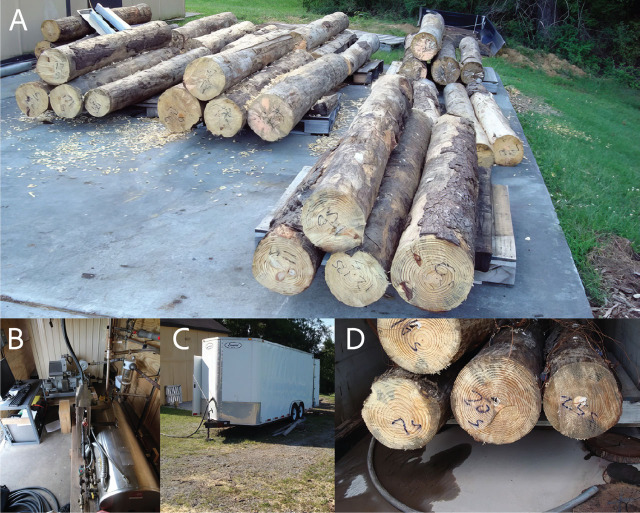
A) Whole yellow pine (*Pinus* spp.) logs were debarked at the woodlot and shipped to the research lab at Virginia Tech, where they were cut into 2.3 m lengths, and groups of four were placed on wooden pallets and randomly selected for the various treatments. B–D) The vacuum treatment set-up used in this research was custom-built inside a mobile trailer with a vacuum container, and the steam generator was used as the test chamber with the capacity to treat 4 whole 2.3 m logs at the same time. Temperature sensors were attached to the log at the same time at various sites on the log and inside the chamber.

The logs were cut into 2.5 m lengths and treated with a combination of vacuum and steam to evaluate this treatment’s ability to quickly kill pinewood nematodes in naturally infected pine logs used for export (Fig. 2A). A custom-built vacuum chamber (1.5 × 1.5 × 3.0 m capacity) contained a treatment system with an 80Kw electric boiler (Reimers Electra Steam®, Clear Brook, VA) and a 5 hp dry screw vacuum pump (Busch® LLC, Virginia Beach, VA) (Figs. 2B–D). Omega K type thermocouples were used to monitor temperature and to measure energy consumption. Real-time temperature profiles within 3 logs during treatment were acquired from the thermocouple probes and recorded (Fig. 4). Energy usage for each test was measured using a line-powered ELITE pro-XC® (Dent Instruments, Bend, OR) power meter. The mobile system was capable of maintaining a continuous vacuum of less than 5 mm of mercury (Hg) which was unhindered by moisture and loss of air from the logs.

### Testing materials

Forty yellow pine 2.5m long logs with small end diameters ranging from 21.25 to 32.5cm were selected for treatment (Table 1). Four pine logs were placed on a pallet for each treatment (Fig. 2A). A thermocouple was placed near the center of the log and was used to determine when the desired temperature was reached and the time that it was maintained. The temperature was also measured at the center of the end grain at a depth of 20cm.

**Table 1:** The experimental design with schedules of treatments for the eradication of pinewood nematodes, (*Bursaphelenchus xylophilus* (Steiner and Buhrer, 1934) Nickle, 1971), in naturally infested whole pine (*Pinus* spp.) logs.

**Species**	**Treating schedule**	**Temperature target location**	**Number of logs/tests**	**Number of tests**	**Total number of logs**
Pine	48°C/15min.	Center	4	3	12
Pine	56°C/30min.	Center	4	3	12
Pine	60°C/60min.	Center	4	3	12
Control			4		4

The tests took place over a fifteen-day period from Aug. 26 through Sept. 9, 2022, with outside temperatures ranging from 10 to 25°C (Table 2). The time to achieve the vacuum varied from 9 to 11 min., and the steam exposure time was between 311 and 634 min. Total time per treatment ranged between 382 and 675 min., and energy usage varied between 41.5 and 84.7 kwh. The range of these metrics depended mostly on the treatment schedule.

**Table 2:** Testing dates, times, temperatures, and energy usage for the treatment of naturally infested whole pine (*Pinus* spp.) logs with pinewood nematodes (*Bursaphelenchus xylophilus* (Steiner and Buhrer, 1934) Nickle, 1971).

**Test**	**Date**	**Outside temp.**	**Vacuum time**	**Steam time**	**Holding time**	**Total time**	**Energy usage**

	**2021**	**°C**	**min**	**min**	**°C/min**	**min**	**kwh**

**Pine**							
1	Aug. 26	21	10	391	48/15	416	49.7
2	Aug. 30	22	9	464	48/15	488	58.4
3	Aug. 31	25	10	522	56/30	562	62.0
4	Sept. 1	22	11	311	60/60	382	41.5
5	Sept. 2	18	11	634	56/30	675	84.7
6	Sept. 3	16	10	598	60/60	668	74.2
7	Sept. 7	15	9	532	60/60	601	70.0
8	Sept. 8	21	9	511	56/30	550	62.7
9	Sept. 9	10	10	458	48/15	483	70.6

### Nematode samples

**Figure 3:**
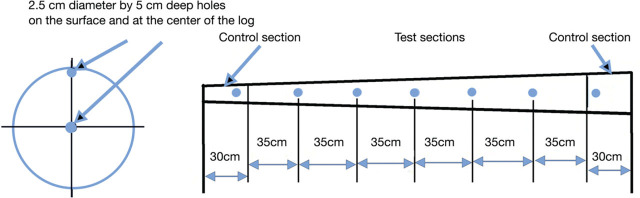
Diagram of the sampling pattern for whole logs includes two sections to serve as controls to determine the presence of pinewood nematodes (*Bursaphelenchus xylophilus* (Steiner & Buhrer, 1934) Nickle, 1971) before treatment and 10 additional sites for sampling after treatment, including 5 near the surface and 5 at the center of the log. Each sample was obtained by drilling a 2.5 cm hole with a 2.5 cm diameter Forstner drill bit. The wood chips from each hole were placed in a plastic bag and stored in a cooler over ice until they were further processed.

After treatment, and when the logs were cool enough to handle, ten samples from each log were taken with a 2.5 cm diameter Forstner drill bit; five were equally spaced on the surface, and five deeper in the same hole, at the center of the log (Fig. 3). Fresh wood chips from the sample cores were stored in sealed plastic bags in the cold-room (8–15°C) until they were extracted via a Baermann funnel. The lab processed approximately 40 samples per day. Funnels were filled with room-temperature tap water and covered with single-ply from an additive-free facial tissue. Ten grams of fresh, wet-weight wood chips were placed on the tissue on the funnel, and the tissue was folded over the top of the funnel to prevent the wicking of the water. Twenty-four hours later, the nematodes were drawn off from the tube from the neck of the funnel and counted in a nematode counting dish with an Olympus inverted light microscope at 40×.

**Figure 4:**
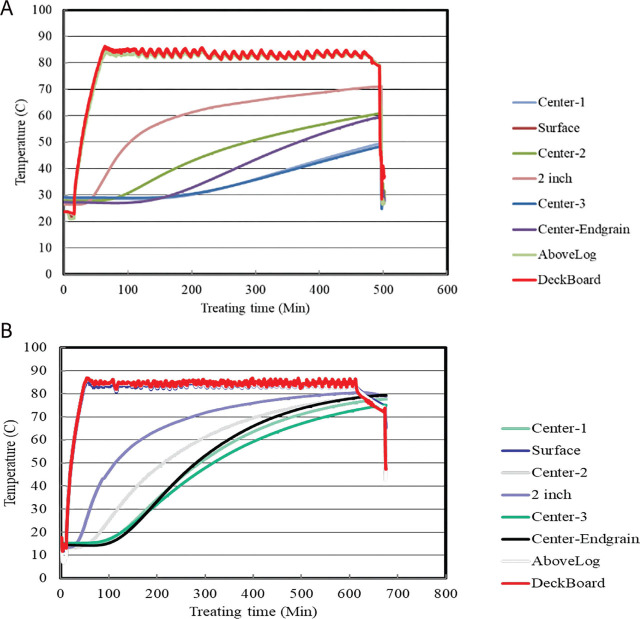
A) Line graph of temperatures at various levels in and on logs and within the chamber during the treatment of 60°C for 60 min. B) Line graph of temperatures at various levels in and on logs and within the chamber during the treatment of 48°C for 15.

The counting dishes were clear plastic pill boxes (2.5cm × 7.5cm) engraved into eight lanes, each the width of the field of view. The entire dish was examined, and all nematodes were identified based on morphological details, including stylet shape, median bulb shape, esophageal gland lobe position, female reproductive system, male spicule shape, and the shape of the male tail.

Most specimens were *Bursaphelenchus xylophilus*; However, occasionally a few Rhabditid specimens were encountered. Rhabditids are commonly found mixed together with the pinewood nematodes so these nematodes were included in all of the counts because they were considered to be survivors. The average number of nematodes per gram of wood tissue for each treatment was determined for the center, surface, and total samples (Table 3). The variation in the results was tested by estimating the standard error of the means with a simple analysis of variance. The variation was small in the treatments (0.5 to 21.0) but high in the controls (120.4 to 131.1), probably because the number of nematodes was low in the treated logs but higher in the untreated logs.

**Table 3:** Means and standard deviations of pinewood nematodes (*Bursaphelenchus xylophilus* (Steiner and Buhrer, 1934) Nickle, 1971) counts per gram of wood shavings from two depths and three treatments of naturally infested whole pine (*Pinus* spp.) logs.

**Treatment schedule**	**Center**		**Surface**		**Total**	

	**Average nematodes/g**	**Std. dev.**	**Average nematodes/g**	**Std. dev.**	**Average nematodes/g**	**Std. dev.**
48°C/15min	2.5	11.3	0.4	0.5	2.9	0.9
56°C/30min	3.9	21.0	0.2	0.8	4.1	19.3
60°C/60min	0^*^	0	0^*^	0	0^*^	0
Control	77	120.4	139.7	131.1	108	142.2

* The average number of nematodes per gram of wood was zero; however, some samples contained at least 1 surviving nematode (4 out of 60 surface and 9 out of 60 center samples).

## Results

Most samples contained only pinewood nematodes. All of the samples with just a few survivors were identified individually from morphological characters. Rhabditids did not occur in any of the samples that had less than 10 nematodes. When more than 10 nematodes were found in a sample, the number of nematodes was divided by 10 to report the number of nematodes per gram of wood. If less than 10 nematodes were found in the 10g sample, it was reported as 1. In reality, the range for this value was between 0.1 and 1. Nevertheless, at least 1 nematode survived the treatment (Tables 3,4). Control logs (n = 4) that were not treated, contained an average of 173 nematodes per g of wood with more in the surface samples of 244/g (n = 20) and less in the center, 102/g (n = 20); however, the number varied from 0/g in one center sample to 614/g in a sample from the surface. Considering the fact that one log weighs approximately 115kg, if there was 1 survivor per g on average, then one log could contain more than 100,000 nematodes compared to the untreated log that contained more than 7 million individuals.

**Table 4:** Numbers of failures for each treatment on the surface and in the center of naturally infested whole pine (*Pinus* spp.) logs for the eradication of pinewood nematodes, (*Bursaphelenchus xylophilus* (Steiner and Buhrer, 1934) Nickle, 1971).

**Treatment**	**Sample location**	**Number**	**Failures**	**Percent failure**
48°C/15min	Surface	60	22	36.7
48°C/15min	Center	60	24	40.0
48°C/15min	Total	120	46	38.3
56°C/30min	Surface	60	8	13.3
56°C/30min	Center	60	33	55.0
56°C/30min	Total	120	41	34.2
60°C/60min	Surface	60	9	15.0
60°C/60min	Center	60	4	6.7
60°C/60min	Total	120	13	10.8
Total	Surface	180	39	21.7
Total	Center	180	61	33.9
Total	Total	360	100	27.8
Control	Surface	20	0	0.0
Control	Center	20	1^*^	5.0
Total	Total	40	1^*^	2.5

* One out of 40 samples from untreated control logs did not contain nematodes and was thus, a failure to serve as a control.

In addition, control samples were collected before treatment from the logs that were treated, from the surface (n = 100), and the center (n = 100). The average number of nematodes per sample was 108/g with a smaller number in the center, 77/g, and more at the surface, 140/g. They varied from 0 to 556.8/g in the center to 1.2 to 566.4/g on the surface. If the nematodes were equally spread throughout the wood, an individual log could contain more than 7 million individuals.

One month later, logs from the 60°C/60 min. that were initially negative were resampled (Table 5). In total, the nematode population increased by 5,381%, with a 2,037% increase at the center and a 12,214% increase in the surface samples.

**Table 5:** Percent increase pinewood nematode (*Bursaphelenchus xylophilus* (Steiner and Buhrer, 1934) Nickle, 1971) populations in naturally infested whole pine (*Pinus* spp.) logs from the 60°C/60 min treatment 1 month after treatment from the surface and center sampling locations.

**Sample**	**Initial**	**1 month later**	**Percent increase**
Surface	0.14	17.1	12,214%
Center	0.27	5.5	2,037%
Total	0.21	11.3	5,381%

One year later, logs that were negative for nematodes in all three protocols were resampled to determine the state of the pinewood nematode population (Table 6). Nematodes were extracted and counted using the same method that was used in the original test. Out of caution, because nematodes from infested wood can readily move into adjacent wood, the wooden pallets that the logs were mounted on for the duration of the tests, were sampled for the occurrence of pinewood nematodes as well. One year later, all of the samples from the treated logs contained nematodes, including those that were initially zero.

**Table 6:** Means of pinewood nematodes (*Bursaphelenchus xylophilus* (Steiner and Buhrer, 1934) Nickle, 1971) counts per gram of wood shavings from treated naturally infested whole pine (*Pinus* spp.) logs one year after treatment.

**Sample number**	**Log number**	**Treatment**	**Nematodes/gram After treatment**	**Nematodes/gram 1 yr. later**
1	16 Surface	48°C/15 min	1	7.9
2	16 Center	48°C/15 min	0	10.4
3	21 Surface	56°C/30 min	0	4.3
4	21 Center	56°C/30 min	1.6	4.3
5	3 Surface	60°C/60 min	0^*^	7.3
6	3 Center	60°C/60 min	0^*^	27

* The average number of nematodes per gram of wood was zero, however, some samples contained at least 1 surviving nematode (4 out of 60 surface and 9 out of 60 center samples).

Treatments at 48°C/15 min. had the most survivors in all of the 12 logs tested. There were 22 surface samples with survivors and 24 center log samples with survivors. Logs that were treated at 56°C/30 min. had the second-most number of survivors, with two out of 10 logs nematode-free; however, 41 samples contained survivors, with 8 from the surface and 33 from the center of the log. From the treatment 60°C/60 min., 5 logs were nematode-free, but 7 logs contained at least one survivor. Altogether, there were 13 samples with nematodes, 4 from the surface samples and 9 from the center of the log. All logs with a maximum diameter larger than 27.9 cm had at least one sample with at least one surviving nematode. In the repeat samples that were collected one month after treatment, all of the samples had at least 1 surviving nematode and up to 227 nematodes per gram. Three of the four pallets were negative, but at least one pinewood nematode was recovered from one pallet, perhaps demonstrating that nematodes from infested wood can migrate to non-infested wood. None of the treatment schedules degraded the quality of the wood.

Energy consumed for each treatment (Table 2) varied from 41.5 to 84.7kWh. The three treatments of 48°C for 15 min ranged from 49.7 to 70.6kWh, and treatments of 56°C for 30 min. varied from 62.0 to 85.7kWh, and treatments of 60°C for 60 min. ran from 41.5 to 74.2kWh. Vacuum time varied from 9 to 11 min. and steam time varied from 311 to 634 min. The amount of energy used may be related to the outside temperatures which varied from 10 to 25°C. The 56°C for 30 min treatment consumed the most energy (69.8kWh) and took the most time (596.3 min.), even though the average outside temperature for the three days of treatment was the highest (21.3°C). Treatments of 48°C for 15 min. used slightly less energy and time (52.1 vs 61.9kWh and 462.3 vs 550.3 min.) than the 60°C for 60 min. treatment and the average outside temperature for both treatments was the same (17.6°C).

## Discussion

The pinewood nematodes were not completely killed even at the hottest and longest schedule of 60°C/60 min. because at least one individual survived in one or more of the logs. However, higher temperatures and longer times of exposure increased mortality. Although the statistical average was zero in the 60°C for 60 min treatment, there were survivors in several of the logs, and one month after treatment, all of the logs tested, contained living nematodes. There is some evidence in the literature of pinewood nematodes surviving high temperatures. Smith (1991) in a survey of the literature showed survivors after heat treatment of 5 mm thick pine, spruce, and hemlock specimens at 55°C and 60°C, held for 15 minutes. Qi Longjun *et al*. (2005) also showed survivors in wood treated to 56°C and held for 30 minutes. *Bursaphelenchus xylophilus* has been shown to survive 45°C for 100 min, but all specimens were dead after 210 min (Xie *et al*. 2009). It should be noted that the heat treatments significantly reduced the nematode population, however, the researchers reported there were survivors found shortly after treatment in many samples. In these experiments there is no indication that post treatment propagation of survivors was monitored. Furthermore, the cross-section thickness of the wood specimens used in these tests were significantly less than the size of whole logs (Dwinell 1990). Nematode populations in infected logs can be in the multiple millions. Even Probit 9 efficacy levels imply 70 survivors in an infected log with 7 million nematodes. Each surviving *Bursaphelenchus xylophilus* female will produce 50 to 500 adults in four days, so after one month, the still green log may have between 25,000 to 250,000 adult nematodes. International supply chains for logs are often longer than 30 days. So, Probit 9 would not exist when logs arrive at their destination.

For log diameters of less than 33 cm, the total time to reach the highest temperature of 60°C was 668 min, whereas the most energy consumed was 84.7kWh. At an average rate of 14 cents/kWh, this treatment would cost $11.86 for 4 logs that were 2.5m long. Increasing the time and/or temperature of the treatment will increase the cost significantly. Fortunately, log quality (color and end splits, etc.) was not affected by the vacuum steam treatment.

Although the 60°C/60 min treatment reduced the pinewood nematode population to a statistical mean of zero, it did not eradicate all of the nematodes. Furthermore, after one month, the logs with no detectable nematodes after treatment had increased to detectable levels, and all logs tested one year later still had living nematodes. Therefore, none of the treatments were successful in reaching the goal of zero nematodes or even a Probit 9 level of morbidity.

The question is, how were the survivors in our experiments able to survive 60°C for 60 min? At least two possibilities exist: 1) most likely, some areas within the log were somehow insulated from the heat and did not reach the lethal temperature, or 2) less likely, perhaps dauer larvae, or some other survival stage, were able to survive temperatures that are normally deadly. For example, the seed gall nematode, *Anguina agrostis* (Steinbuch, 1799) Filipjev, 1936, survived 155°C for 5 min in an anhydrobiotic state (Eisenback *et al*. 2013). Also, the fact that there were more survivors in the samples near the surface of the log (n=9) than in the center (n=4) supports the survival stage hypothesis rather than that of insulation. Perhaps a better temperature monitoring profile will be required to achieve the desired goal of eradicating pinewood nematodes from whole logs.

This also calls for a discussion about efficacy levels and when they should be measured after treatments, given the speed with which surviving nematodes propagate. If the goal is to reduce the transport between countries of nematodes in logs passing through month-long supply chains, what are acceptable levels of infestations at the destination?
